# Circ_0000182 promotes cholesterol synthesis and proliferation of stomach adenocarcinoma cells by targeting miR-579-3p/SQLE axis

**DOI:** 10.1007/s12672-023-00630-5

**Published:** 2023-02-20

**Authors:** Cui-juan Qian, Yu-xin Zhou, Lin-ken Wu, Yi-chao Wang, Xiao-sheng Teng, Jun Yao

**Affiliations:** 1 Early Gastrointestinal Cancer Research Center, Taizhou Central Hospital (Taizhou University Hospital), Taizhou University, Taizhou, 318000 Zhejiang Province China; 2 School of Medicine, Taizhou University, Taizhou, 318000 Zhejiang Province China; 3 Department of Medical Laboratory, Taizhou Central Hospital (Taizhou University Hospital), Taizhou University, Taizhou, 318000 Zhejiang Province China; 4 Department of Gastroenterology, Taizhou Central Hospital (Taizhou University Hospital), Taizhou University, Taizhou, 318000 Zhejiang Province China

**Keywords:** STAD, Cholesterol metabolism, circ_0000182, miR-579-3p, SQLE

## Abstract

**Background:**

Circular RNAs (circRNAs) or cholesterol metabolism have been demonstrated to participate in stomach adenocarcinoma (STAD) progression. However, the relationship between circRNAs and cholesterol metabolism in STAD and its underlined mechanism remain unclear.

**Methods:**

RNA and protein expression levels were detected by qRT-PCR and Western blot. Cell proliferation was assessed by CCK-8, EdU incorporation and colony formation assays. Total cholesterol (TC) and free cholesterol (FC) levels were measured by the corresponding kits. The relationships between circ_0000182 and miR-579-3p or squalene epoxidase (SQLE) mRNA were investigated by bioinformatics analysis, RNA-RNA pull-down, luciferase reporter and RIP assays.

**Results:**

We found that circ_0000182 expression was significantly up-regulated in both STAD tissues and cell lines, and high circ_0000182 expression was correlated with increased tumor size. Circ_0000182 promoted cell proliferation and cholesterol synthesis of STAD cells. Accordingly, cell proliferation, cholesterol synthesis and SQLE expression were significantly inhibited by circ_0000182 knockdown in STAD cells, and these effects were partly reversed by miR-579-3p inhibition or SQLE over-expression. Furthermore, we identified that circ_0000182 acted as a competing endogenous RNA (ceRNA) by sponging miR-579-3p, thereby facilitating SQLE expression, cholesterol synthesis and cell proliferation.

**Conclusion:**

Circ_0000182 promotes cholesterol synthesis and proliferation of STAD cells by enhancing SQLE expression via sponging miR-579-3p.

**Supplementary Information:**

The online version contains supplementary material available at 10.1007/s12672-023-00630-5.

## Introduction

According to the latest data released by the International Agency for Research on Cancer (IARC) of the World Health Organization (WHO), stomach cancer ranks the fifth in the incidence rate and fourth in tumor related mortality rate of malignant tumors worldwide [1, 2]. The main pathological type of stomach cancer is stomach adenocarcinoma (STAD), which accounts for over 95% of all stomach cancer cases [3]. Because of the lack of specific early symptoms and diagnostic markers, most patients with STAD are diagnosed at the advanced-stages, which leads to the high mortality rate of patients with STAD [4–6]. Despite recent advances in diagnosis and treatment of patients with STAD, the overall survival rate (OSR) in the patients has not markedly elevated over the last decade [7, 8]. Therefore, the searches for specific tumor markers and effective therapeutic targets have become focuses in STAD diagnosis and treatment fields in recent years.

Abnormal tumor cholesterol metabolisms are mainly manifested by up-regulation of cholesterol synthesis, increase of cholesterol uptake, and abnormal accumulation of a large number of metabolic products, which enhance the adaptability of tumor cells to the microenvironment and promote the cell proliferation, invasion, metastasis and other malignant biological behaviors [9–12]. A report published in the “Blood” in 2022 showed that cholesterol metabolism provides indispensable cell membrane components and many important metabolic intermediates to support cell proliferation in hematopoietic malignancies [9]. Squalene epoxidase (SQLE) is a monooxygenase that catalyzes squalene to 2,3-epoxy squalene in cholesterol synthesis pathway [13–15]. Moreover, SQLE has been found to be over-expressed in breast cancer and hepatocellular carcinoma tissues, and associated with poor prognosis of patients with these tumors [13–15]. However, the role and mechanism of SQLE-mediated cholesterol synthesis in STAD progression remain further explored.

Increasing studies in recent years demonstrated that non-coding RNAs (ncRNAs), including circular RNAs (circRNAs) and microRNAs (miRNAs), may function as tumor suppressors or oncogenes in various type of tumors [16]. Furthermore, some circRNAs have been identified as key regulators in STAD progression, and may be used as tumor biomarkers for early diagnosis, therapy and prognosis of STAD [17]. As reported, circRNAs are involved in lipid metabolism, and function as crucial regulators in the occurrence and development of gestational diabetes mellitus and cardiovascular disease (CVD) [18, 19]. Furthermore, some circRNAs have been shown to participate in cholesterol metabolism by reducing cholesterol efflux from macrophages, and promoting cholesterol synthesis in aortic endothelial cells [20, 21]. However, the role and mechanism of circRNAs in tumor cholesterol metabolism still remain to be explored. Therefore, exploring the role and mechanism of circRNAs in STAD cholesterol metabolism will help clarify the relationship between cholesterol metabolism and STAD progression from the perspective of ncRNAs.

It's worth noting that circRNAs can function as competing endogenous RNAs (ceRNAs) to sponge their corresponding miRNAs and thus regulate their downstream target genes’ expressions in STAD [22]. Interestingly, our previous study demonstrated that circRNA circ_0001093 could act as a ceRNA for miR-579-3p to promote glutamine metabolism and cell proliferation of esophageal squamous cell carcinoma (ESCC) cells [23]. MiR-579-3p has been reported to be down-regulated and exert a tumor suppressor in various types of tumors, including ESCC [23], hepatocellular carcinoma [24], nasopharyngeal carcinoma [25], and pancreatic carcinoma [26]. Based on GEPIA database (http://gepia.cancer-pku.cn/), SQLE was found to be highly expressed in STAD tissues. MiRNA target prediction databases TargetScanHuman (https://www.targetscan.org/) and miRDB (https://mirdb.org/) indicated SQLE mRNA possessed a miR-579-3p (hsa-miR-579-3p) targeting binding sequence. And then circRNA target prediction databases Circular RNA Interactome (https://circinteractome.nia.nih.gov/) and circbank (http://www.circbank.cn/) indicated miR-579-3p possessed a circ_0000182 (hsa_circ_0000182) targeting binding sequence. Therefore, it is reasonable to speculate that circ_0000182/miR-579-3p/SQLE axis may participate in regulating STAD progression.

Circ_0000182 is a newly discovered circRNA with a spliced sequence length of 458 bp, and the coding gene is located at chr1:213037066–213058738 (https://circinteractome.irp.nia.nih.gov/). However, so far, the role of circ_0000182 in tumors has not been reported. Through bioinformatics analysis and experimental validation, we found that circ_0000182 could modulate the expression of SQLE through ceRNA mechanism, and play a role in regulating cholesterol metabolism in STAD. Thus, exploring its role and mechanism of circ_0000182 in STAD progression may provide novel biomarkers and/or targets for diagnosis and treatment of STAD.

## Materials and methods

### Clinical samples

Clinical studies were approved by the Ethics Committee of Taizhou University Hospital. Forty patients with STAD at Taizhou University Hospital were recruited and signed informed consent voluntarily. Major inclusion criteria were: (1) Patients diagnosed with STAD by two senior pathologists; (2) Patients provided informed consents. Major exclusion criteria were: (1) Patients received chemotherapy or other therapies before the surgery; (2) Patients with a history of other malignant diseases within 5 years. A total of primary STAD and matched normal tissue specimens (Taizhou cohort 1 N = 40 for qPCR and Western blot; Taizhou cohort 1 N = 78 for IHC) were obtained from the Taizhou Central Hospital (Taizhou University Hospital) from 2016 to 2020. STAD tissue specimens and their matched non-tumor tissue specimens were immediately frozen in liquid nitrogen.

### Cell culture

Human gastric mucosal epithelial cells (GES-1) and human STAD cell lines (MKN-28, AGS, SGC-7901 and BGC-823) were obtained from the American Type Culture Collection (ATCC, Manassas, VA, USA). All the cells were incubated in RPMI 1640 medium (Gibco, Carlsbad, CA, USA) mixed with 10% fetal bovine serum (FBS, Gibco) in an atmosphere at 37 ℃ with 5% CO_2_.

### qRT-PCR

Trizol reagent (Invitrogen, Carlsbad, CA, USA) was used for total RNA extractions from STAD tissue specimens and in vitro cultivated cells. Following the determination of RNA concentration and purity, the total RNA was converted to cDNA using the PrimeScript 1st Strand cDNA Synthesis Kit (Takara, Dalian, China) or the Mir-X™ miRNA First-Strand Synthesis Kit (TaKaRa) according to the manufacturer’s requirements, respectively. The obtained cDNA was then analyzed by qRT-PCR using SYBR premix Ex Taq™ II kit (Takara) according to the manufacturer’s requirements. GAPDH and U6 were used as internal controls for mRNAs and miR‐579-3p, respectively. All primer sequences designed using Primer-Blast online tool (https://www.ncbi.nlm.nih.gov/tools/primer-blast/) or as described previously [25, 27] are listed in Table 1. Finally, the relative RNA expression levels were evaluated using the 2^−ΔΔCt^ method.

**Table 1:** qRT-PCR primer sequences

Name	Primer sequence
circ_0000182	Forward: 5′-TGCTGGAAGGATTGGGCTAA-3′ Reverse: 5′-CAAGCACCCCTCCAGTAACA-3′
miR-579-3p	Forward:5′-GCGCTTCATTTGGTATAAACC-3′
	Reverse:5′-GGCAATTGCACTGGATG-3′
SQLE	Forward: 5′-TGGTTACATGATTCATGATC-3′ Reverse: 5′-TACTGAACTCCCATCACAAC-3′
GAPDH	Forward: 5′-GCACCGTCAAGGCTGAGAAC-3′ Reverse: 5′-GCCTTCTCCATGGTGGTGAA-3′
U6	Forward: 5′-GCTTCGGCAGCACATATACTAAAAT-3′ Reverse: 5′-CGCTTCACGAATTTGCGTGTCAT-3′

### RNase R digestion assay

To identify the character of circ_0000182, 5 μg total RNA isolated from STAD cells was subjected for RNase R (Tiangen Biochemical, China) for 30 min at 37 ℃. Subsequently, the obtained total RNA was analysed by qRT-PCR.

### CCK-8 assay

The CCK-8 kit (Abcam, Cambridge, MA, USA) was used following the manufacturer’s introductions. In detail, STAD cells (3 × 10^3^/100 μl/well) were seeded into the 96-well plates, and six replicates for each sample were set at the same time. Different intervention factors and time were applied to the STAD cells, and then 10 μl of CCK-8 solution which was evenly mixed in 100 μl of fresh medium were added into each well. Plates were incubated for 2 h at 37 ℃, and the absorbance was recorded at 450 nm with a microplate reader (Bio-Rad, Hercules, CA, USA).

### Colony formation assay

STAD cells were plated at a density of 40 cells/cm^2^ in 6-well plates and incubated for 14 days. The medium were changed every 3 days. Subsequently, the resulting cell colonies were washed thrice with PBS, fixed with 4% paraformaldehyde for 10 min at 4 ℃, and stained with 0.1% crystal violet for 30 min at room temperature. Visible colonies (> 50 cells/colony) were manually counted.

### EdU incorporation assay

The cell proliferation was detected using a BeyoClick™ EDU Cell Proliferation Kit with 3,3',5,5'-Tetramethylbenzidine (TMB) (Beyotime Biotechnology, Shanghai, China) according to the manufacturer’s instructions. In brief, STAD cells were seeded in 96-well plates at 5 × 10^3^ cells per well. 5-ethynyl-2'-deoxyuridine (EdU) was incorporated into the DNA synthesis of cells. Under the catalysis of copper ion, EdU was labeled with biotin, and then HRP streptavidin was added to combine with biotin, and the color was determined by TMB chromogenic solution. The plates were read with a single wavelength of 630 nm using a microplate reader (Thermo Scientific, Rochester, NY, USA).

### Total cholesterol (TC) and free cholesterol (FC) assays

The amounts of TC and FC were assessed with a TC quantification detection kit (Solarbio, Beijing, China) and a FC quantification detection kit (Solarbio) following the manufacturers’ instructions, respectively. In brief, the culture medium was discarded from 12-well plates and the cells were washed twice with PBS. 0.5 ml cell lysate was added to every 5 × 10^6^ cells, and 0.5 ml distilled water was added after severe oscillation. After 12,000 × g centrifuged for 10 min at 4 ℃, the lower supernatant was collected. After adding the trichloroacetic acid to precipitate cells, the cells were broken up by ultrasound after adding equal volume of chloroform: methanol mixed solution (2:1). After centrifugation, the supernatant was collected and the contents of TC and FC were determined by enzyme colorimetry according to the instructions of the kits. Next, the remaining cell sediment was dissolved in 0.1 mol/L NaOH, and the intracellular protein content was detected by Bradford method. The intracellular cholesterol content is expressed in μg/mg cell protein.

### Western blotting

The whole extracts from BGC-823 cells were prepared using RIPA buffer (Beyotime, Shanghai, China), and the total protein concentrations in the extracts were quantified using a BCA Protein Assay kit (Pierce Rockford, IL, USA) following the manufacturers’ introductions. The protein samples were subjected to sodium dodecylsulphate-polyacrylamide gel electrophoresis (SDS-PAGE), and then transferred from the SDS-PAGE gel to the PVDF membrane (Millipore, MA, USA), which was then incubated overnight at 4 ℃ with primary antibody. The primary antibodies against SQLE (1:1000, catalog no.ab189773; Abcam, Cambridge, MA, USA) and GAPDH (1:1000, catalog no. ab181602; Abcam) were used. GAPDH was used as an internal control. The next day, the PVDF membrane was washed and then incubated with a specific secondary antibody conjugated to horseradish peroxidase for 2 h at room temperature. Finally, immunoreactivity was detected by an enhanced chemiluminescence system kit (Pierce, Waltham, MA, USA) and photographed by an LAS-4000 imaging system (Fujifilm Holdings Corporation, Tokyo, Japan).

### Tissue array

Analysis of SQLE protein expression by immunohistochemistry (IHC) was done on a STAD tissue array (Shanghai Outdo Biotech, Shanghai, China), containing 78 pairs of samples from STAD tissues and adjacent non-tumor tissues, using an automated immunostainer (Benchmark XT; Roche, Basel, Switzerland). Tumor staging was judged by two senior pathologists using the tumor-node-metastasis (TNM) classification system according to the protocol of International Union Against Cancer (UICC). The DAKO EnVision system (DAKO, Carpinteria, CA, USA) was used to analyze the immunohistochemical expression of SQLE protein. All images (× 200) were photographed and analyzed with an Aperio scanner (Aperio Technologies, Vista, CA, USA).

### RNA pull-down assay

48 h after transfection with biotinylated wild-type (WT) miR-579-3p (Bio-miR-579-3p-WT), mutant (MT) miR-579-3p (Bio-miR-579-3p-MT) or antagonistic miR-579-3p probe (GenePharma, Shanghai, China), the STAD cells were collected and lysed in specific lysis buffer (Ambion, Austin, TX, USA) for 10 min, and then mixed with M-280 streptavidin magnetic beads (Sigma-Aldrich, St. Louis, MO, USA) at 4 ℃ for 3 h. Trizol reagent (Invitrogen) was used to elute and purify the interacting RNA complex, and qPCR was used to detect the expression of circ_0000182 in the RNA complex.

### Luciferase reporter assay

In order to generate wild-type miR-579-3p reporter gene (miR-579-3p-WT) and SQLE reporter gene (SQLE-WT), the partial sequences of miR-579-3p and SQLE 3′-untranslated region (UTR), which separately contained the putative circ_0000182 and miR-579-3p binding sites, were amplified by PCR and then cloned into the pmirGLO luciferase vectors (Promega, Madison, WI, USA), respectively. Mutant miR-579-3p (circ_0000182 target site-mutation miR-579-3p, miR-579-3p-MUT) reporter gene and mutant SQLE (miR-579-3p target site-mutation SQLE 3′-UTR, SQLE-MUT) reporter gene were produced by GeneArt™ Site-Directed Mutagenesis System (Thermo Fisher Scientific, Waltham, MA, USA). All constructs were verified by DNA sequencing. Subsequently, the luciferase reporter genes, sh-circ_0000182 or sh-NC, and miR-579-3p mimic or control mimic were co-transfected into STAD cells, respectively. After 48 h, the cells were harvested, and the luciferase activity was detected using the dual luciferase reporter system (Promega).

### RIP assay

After transfection of STAD cells with miR-579-3p mimic or control mimic (Promega, Madison, WI, USA), RIP detection was performed using the Magna RIP RNA-Binding Protein Immunoprecipitation Kit (Millipore, MA, USA) according to the manufacturer's instructions. In brief, the collected cells were lysed in RIP lysis buffer. Then, the cell lysis was incubated with RIP buffer containing magnetic beads linked to Argonaute2 (AGO2) antibody or IgG antibody at 4 ℃, using IgG as a negative control, the RNA complex was extracted after elution with protease K, and qPCR was used to detect the expression of circ_0000182 in the RNA complex.

### Statistical analysis

Data were analyzed using SPSS 25.0 software (SPSS Inc., IL, USA) and presented as mean ± standard deviation (SD). Comparisons of two groups were analyzed by Student's t tests, and comparisons of three or more groups comparisons were analyzed by ANOVA (Tukey's post hoc test). The correlations between circ_0000182 expression and clinical parameters were analyzed using the χ^2^-test. The correlations between circ_0000182, miR-579-3p and SQLE mRNA expressions in STAD tissues were determined by Pearson’s correlation analysis. *P* values < 0.05 were considered to be statistically significant.

## Results

### SQLE and circ_0000182 expressions are both up-modulated, and miR-579-3p expression is down-modulated in STAD tissues

**Fig. 1:**
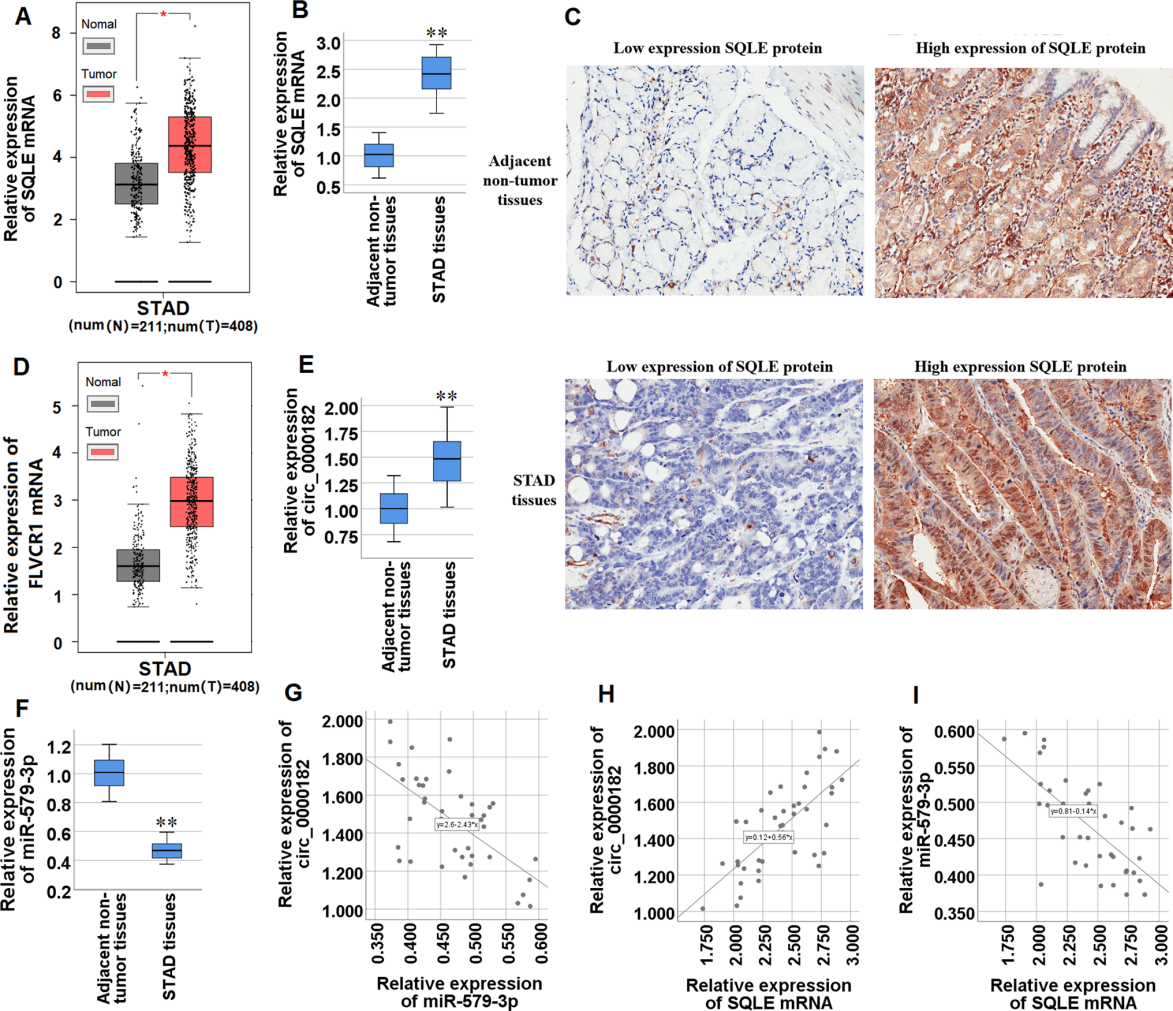
The expression patterns of SQLE, miR-579-3p and circ_0000182 in STAD tissues.** A** The box plot reporter assay reveals the differential expression of SQLE mRNA in 408 STAD and 211 normal tissues in the GEPIA database. **B** qRT-PCR analysis of SQLE mRNA expression in STAD tissues compared with adjacent non-tumor tissues. **C** Immunohistochemical detection of SQLE protein expression in STAD tissues and adjacent non-tumor tissues. The positive staining is brownish yellow and located in the cytoplasm or nucleus (Original magnification × 200). **D** The box plot reporter assay reveals the differential expression of FLVCR1 mRNA in 408 STAD and 211 normal tissues in the GEPIA database. **E** qRT-PCR analysis of circ_0000182 expression in STAD tissues compared with adjacent non-tumor tissues. **F** qRT-PCR analysis of miR-579-3p expression in STAD tissues compared with adjacent non-tumor tissues. **G** Correlations between expression levels of circ_0000182 and miR-579-3p in STAD tissues were analyzed by Pearson's correlation analysis. **H** Correlations between expression levels of circ_0000182 and SQLE mRNA in STAD tissues were analyzed by Pearson's correlation analysis. **I** Correlations between expression levels of miR-579-3p and SQLE mRNA in STAD tissues were analyzed by Pearson's correlation analysis. *P < 0.05, **P < 0.01

To assess the role of SQLE expression in STAD tissues, we acquired its expression profile in 408 STAD tissue samples and 211 normal tissue samples from GEPIA database (http://gepia.cancer-pku.cn/index.html), and found that the mRNA of SQLE was significantly up-modulated in STAD tissues compared with normal tissues (Fig. 1A). Subsequently, we validated SQLE mRNA expression in tissue samples from 40 patients with STAD, and confirmed that SQLE mRNA level was significantly higher in the STAD tissues compared with adjacent non-tumor tissues (Fig. 1B). Further, the results in tissue arrays from 78 STAD patients manifested that the high expression rate of SQLE protein in STAD tissues was 57.69% (45/78), the high expression rate of SQLE protein in adjacent non-tumor tissues was 35.90% (28/78), and there was significant difference between the two groups (*P* < 0.01) (Table 2). SQLE protein was mainly expressed in the cytoplasm and nucleus of STAD tissues, while a small amount was expressed in the cytoplasm and nucleus of adjacent non-tumor tissues (Fig. 1C). Thus, our data demonstrated that SQLE expression was up-regulated in STAD tissues (Figure S1).

**Table 2:** Expression and comparison of SQLE protein in STAD and adjacent non-tumor tissues

Groups	High expression of SQLE protein	Low expression SQLE protein	χ2 value	*P* value
STAD tissues	45	33		
Adjacent non-tumor tissues	28	50	7.440832	0.006376**

^**^P < 0.01

The miRDB database (http://mirdb.org/) was used to predict the miRNAs that bind to the 3'-UTR of SQLE mRNA, and hsa-miR-579-3p (miR-579-3p) with the highest context score (Target Score = 96) was selected as a miRNA that might bind to the 3'-UTR of SQLE mRNA (Table S1), and the TargetScanHuman database (http://www.targetscan.org/) further verified the binding of miR-579-3p to SQLE mRNA 3'-UTR, and the predicted scores (the context +  + score percentile) for the two sites of miR-579-3p binding to SQLE mRNA 3'-UTR both are 93 (Table S2). The circRNAs binding to miR-579-3p were comprehensively analyzed and predicted by Circular RNA Interactome database (https://circinteractome.nia.nih.gov/), hsa_circ_0000182 (circ_0000182) with a context score ≥ 99 was selected as a circRNA that binds to miR-579-3p, and while the binding of circ_0000182 to miR-579-3p sequence was manifested in Table S3. Through GEPIA database analysis, we found that the parental gene of circ_0000182, FLVCR1 (feline leukemia virus subgroup C cellular receptor 1), was significantly highly expressed in STAD tissues (Fig. 1D), which indirectly suggested that circ_0000182 is highly expressed in STAD tissues (Figure S2).

Next, we tested its RNA levels in 40 STAD specimens relative to 40 adjacent non-tumor tissues using qRT-PCR, and found that circ_0000182 expression was considerably increased in the STAD tissues when compared to their matched adjacent non-tumor tissues (Fig. 1E). MiR-579-3p mRNA expression in the 40 specimens of STAD tissues was also examined by qRT-PCR assay, and the result demonstrated that, compared with that found in the paired adjacent non-tumor tissues, miR-579-3p expression level was significantly lower in STAD tissues (Fig. 1F). We further found that circ_0000182 expression level was significantly related to tumor size, but there was no relationship between circ_0000182 expression level with gender, age, HP infection, lymph node metastasis or TNM stage (Table 3). In STAD tissues, circ_0000182 expression manifested a negative and significant correlation with miR-579-3p (r =  − 0.628, *P* < 0.001; Fig. 1G), and a positive and significant correlation with SQLE mRNA (r = 0.706, *P* < 0.001; Fig. 1H). In addition, there was a negative and significant correlation between the miR-579-3p and SQLE mRNA expressions in STAD tissues (r =  − 0.687, *P* < 0.001; Fig. 1I). Taken together, our data suggest that circ_0000182 negatively regulates miR-579-3p expression and positively regulate SQLE expression in STAD tissues, and meanwhile miR-579-3p might negatively regulate SQLE expression in the tissues.

**Table 3:** Relationship between circ_0000182 expression and clinical parameters in 40 clinical STAD tissue samples

Characteristics	Case	Circ_0000182 expression		χ^2^	*P*-value
		High	Low		
Gender				0.416667	0.518605
Male	24	13	11		
Female	16	7	9		
Age (years)				0.102302	0.749085
≤ 65	17	8	9		
> 65	23	12	11		
Lymph node metastasis				2.666667	0.102470
Negative	15	5	10		
Positive	25	15	10		
TNM stage				3.636364	0.056530
I, II	18	6	12		
III	22	14	8		
Tumor size				4.912281	0.026666*
≤ 5 cm	19	6	13		
> 5 cm	21	14	7		
HP infection				0.439560	0.507335
Negative	14	6	8		
Positive	26	14	12		

*P < 0.05

### Circ_0000182 is up-regulated in STAD cell lines, and promotes cell proliferation of STAD cells

**Fig. 2:**
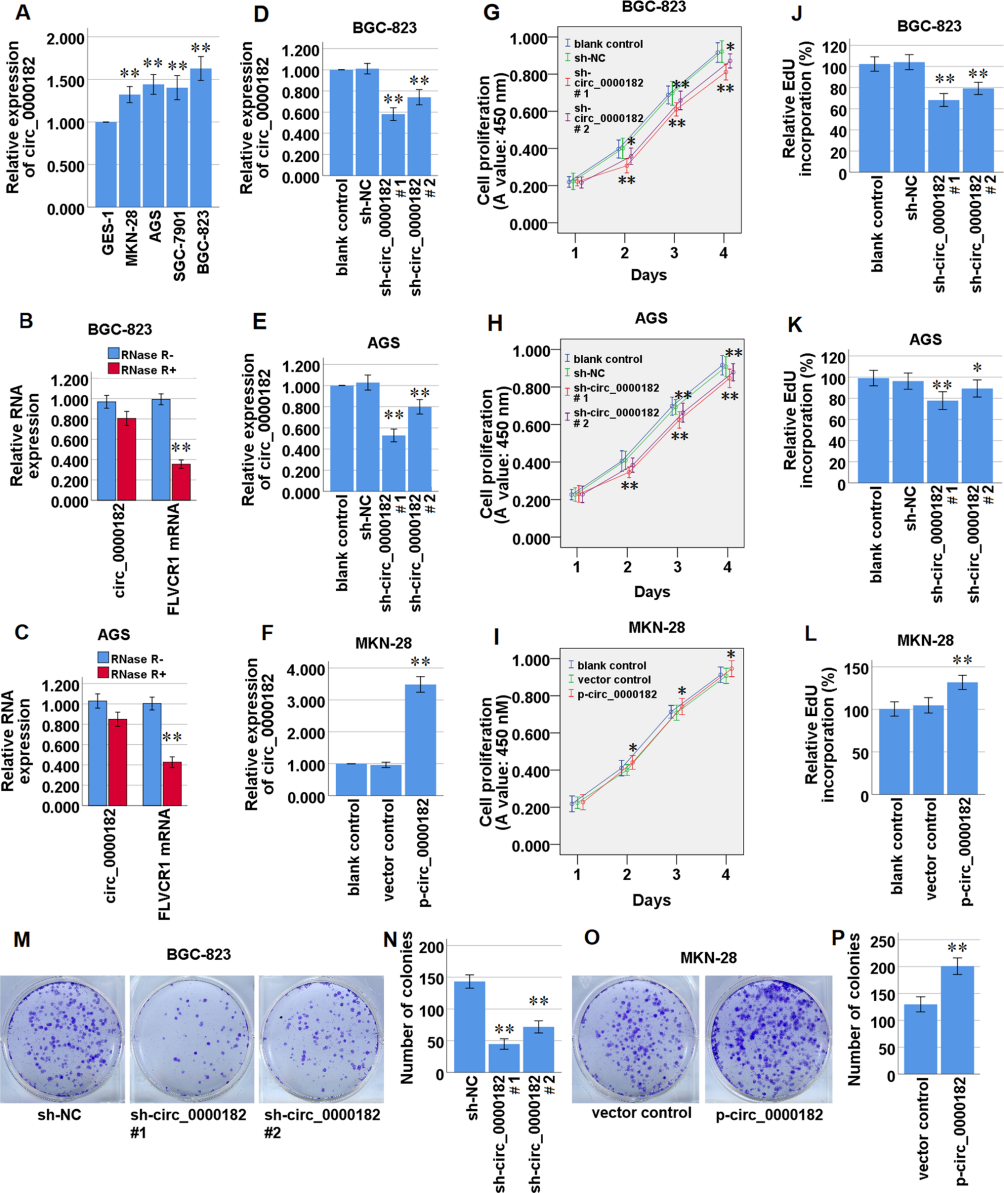
Circ_0000182 expression in STAD cell lines, and effect of circ_0000182 on cell proliferation of STAD cells. **A** The expression patterns of circ_0000182 in GES-1, a normal esophageal epithelial cell line, and STAD cell lines (MKN-28, AGS, SGC-7901 and BGC-823) were detected by qRT-PCR. **B, C** The stability of circ_0000182 was detected by qRT-PCR. RNase R was used to digest circ_0000182 and mRNA of its parent gene FLVCR1. **D, E** The expression of circ_0000182 was down-modulated in BGC-823 and AGS cells transfected with sh-circ_0000182. **F** The expression of circ_0000182 was up-modulated in MKN-28 cells transfected with p-circ_0000182. **G, H** After knockdown of circ_0000182, the cell proliferation of BGC-823 and AGS cells was detected by CCK-8 assay. **I** After over-expression of circ_0000182, the cell proliferation of MKN-28 cells was detected by CCK-8 assay. **J–L** Effect of circ_0000182 on cell proliferation in BGC-823, AGS and MKN-28 cells was detected by EdU incorporation assay. **M–P** Effect of circ_0000182 on cell proliferation in BGC-823 and MKN-28 cells was detected by colony formation assays. Quantitative results of colony numbers are presented in data graphs. *P < 0.05, **P < 0.01

We analyzed the circ_0000182 expression level among STAD cell lines (MKN-28, AGS, SGC-7901 and BGC-823) and human gastric mucosal epithelial cells (GES-1) via qRT-PCR. The results revealed that circ_0000182 expression was significantly up-modulated in the four STAD cell lines compared with GES-1 cells (Fig. 2A). In the four STAD cell lines, BGC-823 and AGS cells manifested the relative high levels of circ_0000182 expression, and meanwhile MKN-28 cells manifested the relative low level of circ_0000182 expression (Fig. 2A). Therefore, the three cell lines were used for the next loss- and gain-of-function experiments. Then, RNase R degradation assay was used to confirm the stable existence of circ_0000182 in STAD cells. The data demonstrated that the circ_0000182 resisted to the RNase R treatment, while the linear RNA (FLVCR1 mRNA) was degraded in both BGC-823 (Fig. 2B) and AGS cells (Fig. 2C).

To further explore the function of circ_0000182 in STAD progression, the short hairpin RNAs (shRNAs) specific against circ_0000182 (sh-circ_0000182#1, sh-circ_0000182#2) and circ_0000182 over-expressing plasmid (p-circ_0000182) were designed and constructed, and circ_0000182 knockdown and circ_0000182 over-expression cell lines were established, respectively. The knockdown or over-expression of circ_0000182 efficiency was evaluated by qRT-PCR (Fig. 2D-F). The CCK-8 assay showed that knockdown of circ_0000182 significantly inhibited the cell proliferation of BGC-823 and AGS cells (Fig. 2G, H), and meanwhile over-expression of circ_0000182 significantly promoted the cell proliferation of MKN-28 cells (Fig. 2I). Similar results were observed in EdU incorporation and colony formation assays. The results of the EdU incorporation assay indicated that compared to the control cells, knockdown of circ_0000182 significantly inhibited the proliferation of BGC-823 and AGS cells, whereas over-expression of circ_0000182 promoted the cell proliferation of MKN-28 cells (Fig. 2J–L). Consistently, compared to the control cells, circ_0000182 over-expression significantly inhibited the clonogenic abilities of BGC-823 cells, while circ_0000182 over-expression manifested the opposite effects in MKN-28 cells (Fig. 2M–P). Taken together, our data demonstrated that circ_0000182 promoted the cell proliferation of STAD cells.

### Circ_000182 participates in promoting cholesterol synthesis in STAD cells

**Fig. 3:**
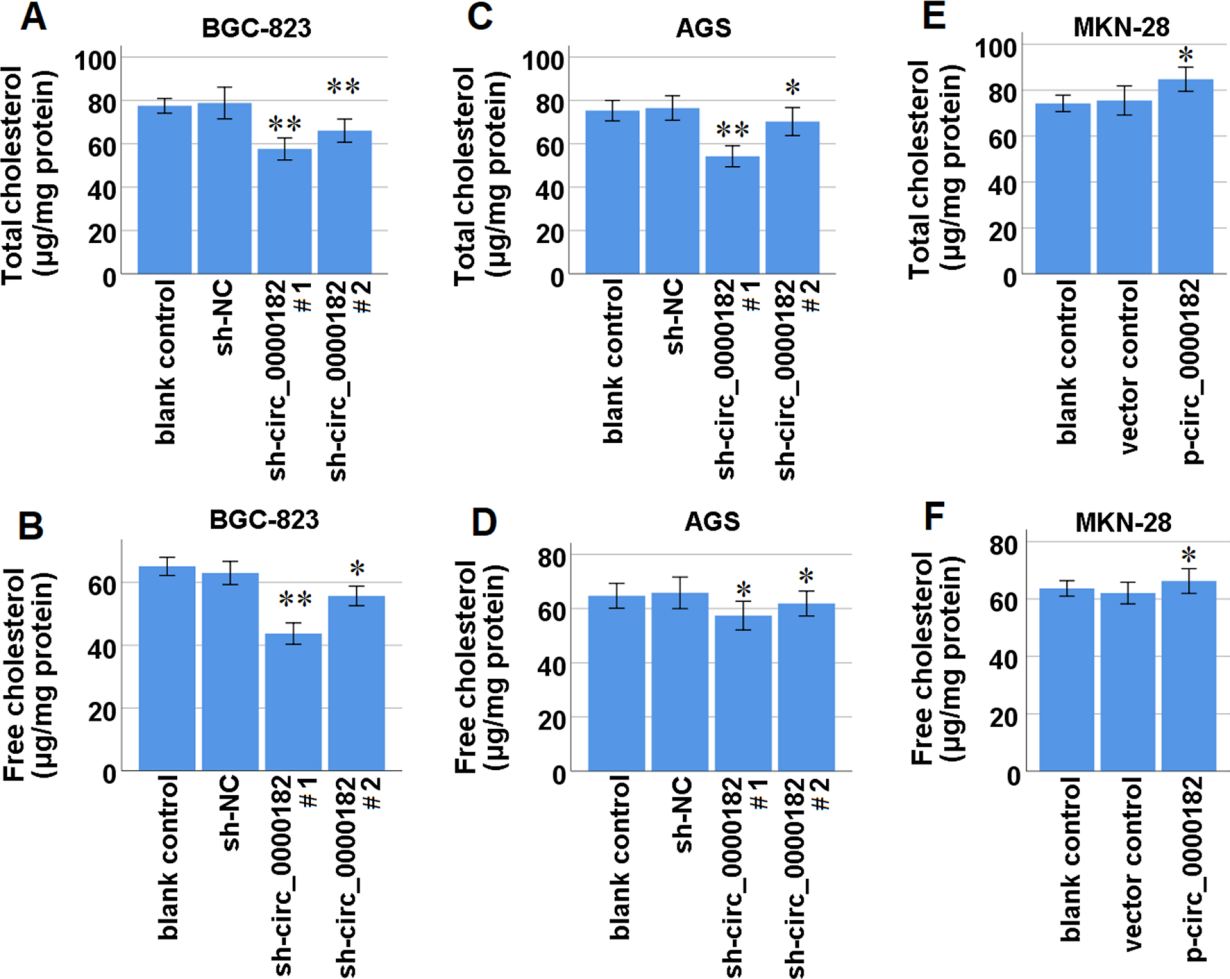
Effects of circ_0000182 on TC and FC levels in STAD cells*.*** A** After knockdown of circ_0000182, TC levels in BGC-823 cells were detected using a total cholesterol quantification detection kit. **B** After knockdown of circ_0000182, FC levels in BGC-823 cells were detected using a free cholesterol quantification detection kit. **C** After knockdown of circ_0000182, TC levels in AGS cells were detected using a total cholesterol quantification detection kit. **D** After knockdown of circ_0000182, FC levels in AGS cells were detected using a free cholesterol quantification detection kit. **E** After over-expression of circ_0000182, TC levels in MKN-28 cells were detected using a total cholesterol quantification detection kit. **F** After over-expression of circ_0000182, FC levels in MKN-28 cells were detected using a free cholesterol quantification detection kit. *P < 0.05, **P < 0.01

To assess whether circ_0000182 contributes to the promotion of cholesterol synthesis in STAD cells, we used the corresponding test kits to detect total cholesterol (TC) and free cholesterol (FC) levels in circ_0000182-knockdown and circ_0000182-over-expression STAD cells. The results demonstrated that circ_0000182 knockdown significantly decreased TC (Fig. 3A and 3C) and FC (Fig. 3B and 3D) levels in BGC-823 and AGS cells. Meanwhile, circ_0000182 over-expression significantly increased TC (Fig. 3E) and FC (Fig. 3F) levels in MKN-28 cells. Taken together, our data indicated that circ_0000182 contributed to the promotion of cholesterol synthesis in STAD cells.

### Circ_0000182 acts as a ceRNA by sponging miR-579-3p in STAD cells

**Fig. 4:**
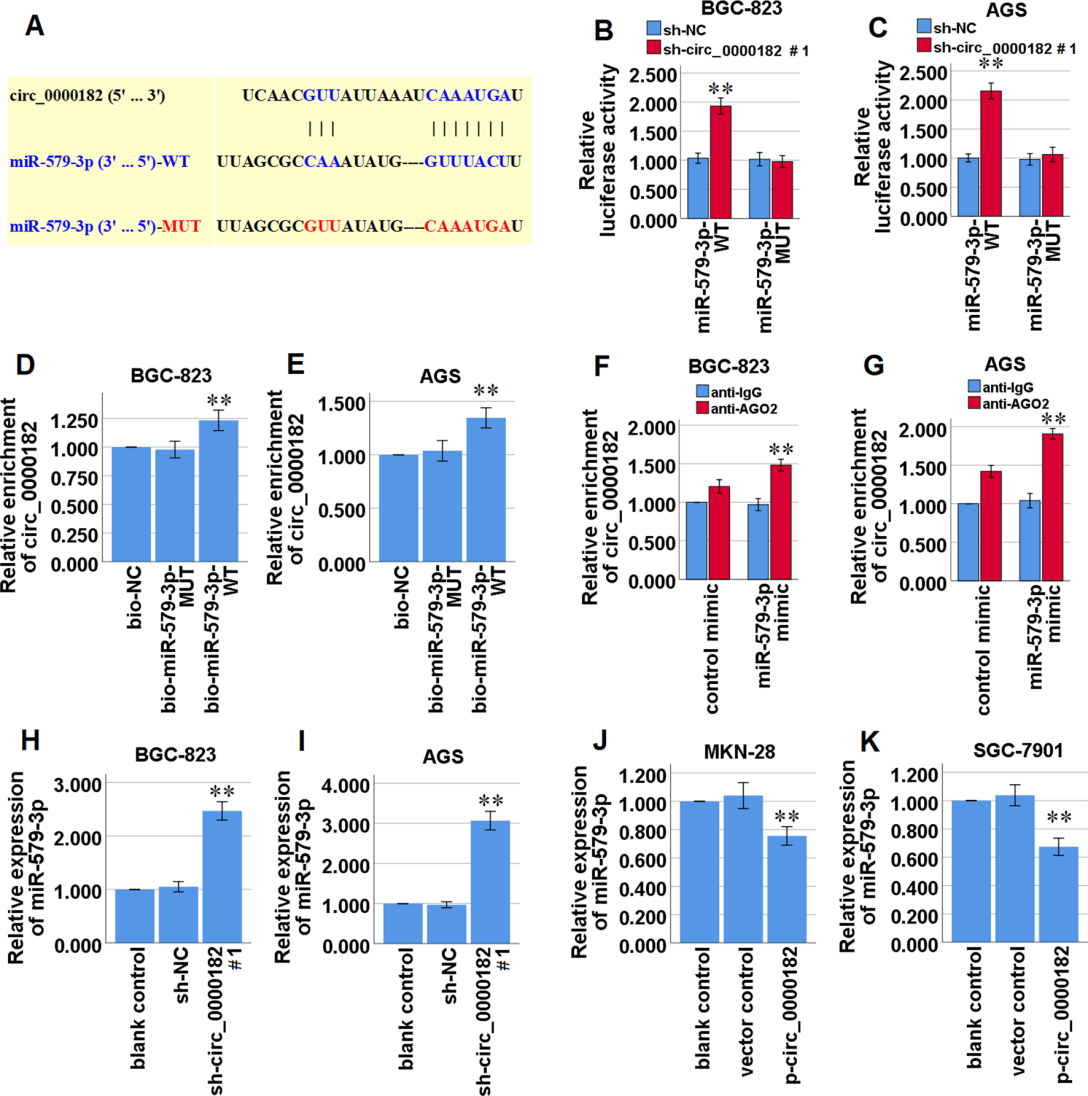
miR-579-3p is targeted and regulated by circ_0000182 in STAD cells. **A** Bioinformatics analysis suggested that circ_0000182 shares the binding sites with miR-579-3p. The wild-type and the mutated sequences of the miR-579-3p (mutation site: red). **B, C** The luciferase activity of BGC-823 and AGS cells was detected in luciferase reporter plasmids containing wild-type miR-579-3p (miR-579-3p-WT) and mutant miR-579-3p (miR-579-3p-MUT) co-transfected with sh-circ_0000182#1 or sh-NC. **D, E** Cell lysate was incubated with biotin-labeled miR-579-3p, and circ_0000182 expression was measured by qRT-PCR in the products of pulldown by biotin-labeled wild-type miR-579-3p (bio-miR-579-3p-WT) or mutant miR-579-3p (bio-miR-579-3p-MUT) in BGC-823 and AGS cells. **F, G** AGO2-RIP followed by qRT-PCR to detect circ_0000182 level in BGC-823 and AGS cells after miR-579-3p over-expression via miR-579-3p mimic. **H, I** qRT-PCR analysis of miR-579-3p expression in BGC-823 and AGS cells transfected with sh-circ_0000182#1 or sh-NC. **J, K** qRT-PCR analysis of miR-579-3p expression in MKN-28 and SGC-7901 cells transfected with p-circ_0000182 or the vector control. *P < 0.05, **P < 0.01

Our present bioinformatics analysis by the Circular RNA Interactome database (https://circinteractome.nia.nih.gov/) manifested that circ_0000182 might share the binding sites with miR-579-3p (Fig. 4A). To investigate the binding capability of circ_0000182 and miR-579-3p, a luciferase reporter assay and a RNA-RNA pull-down assay were conducted. The result of luciferase reporter assay manifested that the luciferase activity of wild-type miR-579-3p was enhanced by circ_0000182 knockdown in BGC-823 and AGS cells, while that of mutant miR-579-3p was not significantly changed in the cells (Fig. 4B, C), and the result of RNA-RNA pull-down assay revealed that wild-type miR-579-3p was captured by circ_0000182 in BGC-823 and AGS cells (Fig. 4D, E). In addition, the RIP assay result presented that both circ_0000182 and miR-579-3p were markedly enriched in anti-Ago2 immunoprecipitation compared with the control mimic group, indicating that circ_0000182 and miR-579-3p coexisted in RNA Induced Silencing Complex (RISC) (Fig. 4F, G).

To further investigate whether circ_0000182 could modulate miR-579-3p expression in STAD cells, we measured the miR-579-3p expression in circ_0000182-knockdown BGC-823 and AGS cells and circ_0000182-overexpression MKN-28 and SGC-7901 cells, and found that knockdown of circ_0000182 significantly promoted miR-579-3p expression in BGC-823 and AGS cells (Fig. 4H, I), and meanwhile over-expression of circ_0000182 significantly inhibited miR-579-3p expression in MKN-28 and SGC-7901 cells (Fig. 4J, K). Taken together, these data confirmed that circ_0000182 targeted miR-579-3p in STAD cells by directly binding miR-579-3p.

### miR-579-3p targets SQLE in STAD cells

**Fig. 5:**
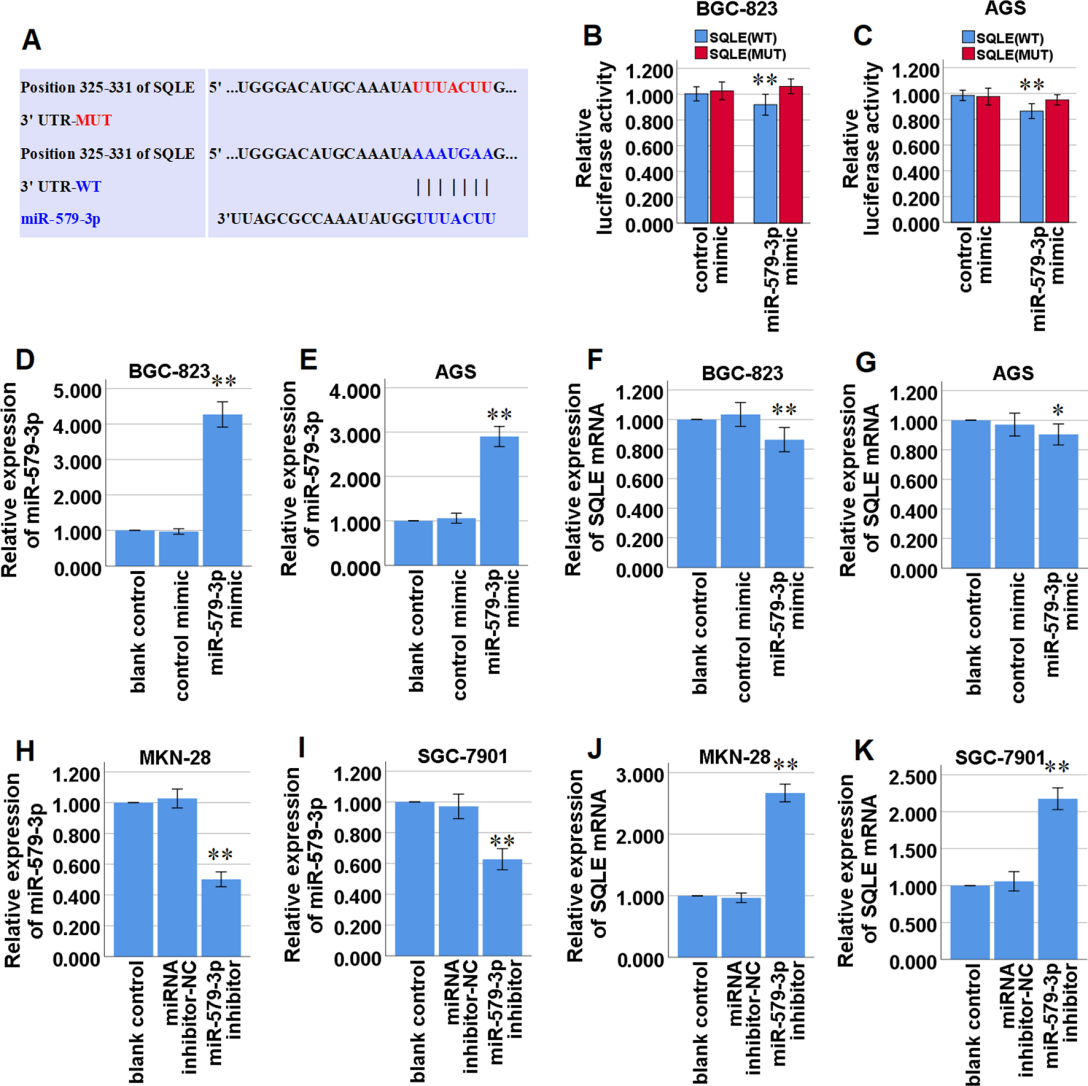
SQLE mRNA was targeted and modulated by miR-579-3p in STAD cells. **A** Bioinformatics analysis suggested that SQLE 3'-UTR shares the binding sites with miR-579-3p. The wild-type and the mutated sequences of SQLE mRNA 3'-UTR (mutation site: red). **B, C** The luciferase activity of BGC-823 and AGS cells was assessed in luciferase reporter plasmid containing SQLE-WT and SQLE-MUT co-transfected with miR-579-3p mimic or negative control (control mimic). **D, E** qRT-PCR analysis of miR-579-3p expression in BGC-823 and AGS cells transfected with miR-579-3p mimic or control mimic. **F, G** qRT-PCR analysis of SQLE mRNA expression in BGC-823 and AGS cells transfected with miR-579-3p mimic or control mimic. **H, I** qRT-PCR analysis of miR-579-3p expression in MKN-28 and SGC-7901 cells transfected with miR-579-3p inhibitor or miRNA inhibitor-negative control (NC). **J, K** qRT-PCR analysis of SQLE mRNA expression in MKN-28 and SGC-7901 cells transfected with miR-579-3p inhibitor or miRNA inhibitor-NC. *P < 0.05, **P < 0.01

Our present bioinformatics analysis by the TargetScan database (https://www.targetscan.org/vert_80/) manifested that miR-579-3p might share the binding sites with SQLE mRNA 3'-UTR (Fig. 5A). A dual luciferase reporter assay was used to confirm that miR-579-3p directly targets SQLE mRNA 3'-UTR in BGC-823 and AGS cells, and demonstrated that the miR-579-3p mimic led to a significant decrease in the luciferase activity of the wild type of SQLE mRNA 3'-UTR (SQLE-WT) reporter in BGC-823 and AGS cells, but not in the mutant 3'-UTR of SQLE (SQLE-MUT) reporter in the cells (Fig. 5B, C). Furthermore, qRT-PCR analysis demonstrated that up-regulation of miR-579-3p expression via miR-579-3p mimic (Fig. 5D, E) significantly decreased SQLE mRNA expression in BGC-823 and AGS cells (Fig. 5F,G). And meanwhile down-regulation of miR-579-3p expression via miR-579-3p inhibitor (Fig. 5H, I) significantly increased SQLE mRNA expression in MKN-28 and SGC-7901 cells (Fig. 5J, K). Taken together, these data confirmed that miR-579-3p targeted SQLE in STAD cells by directly targeting SQLE mRNA 3'-UTR.

### Knockdown of circ_0000182 inhibits cell proliferation and cholesterol synthesis of BGC-823 cells via miR-579-3p/SQLE axis

**Fig. 6:**
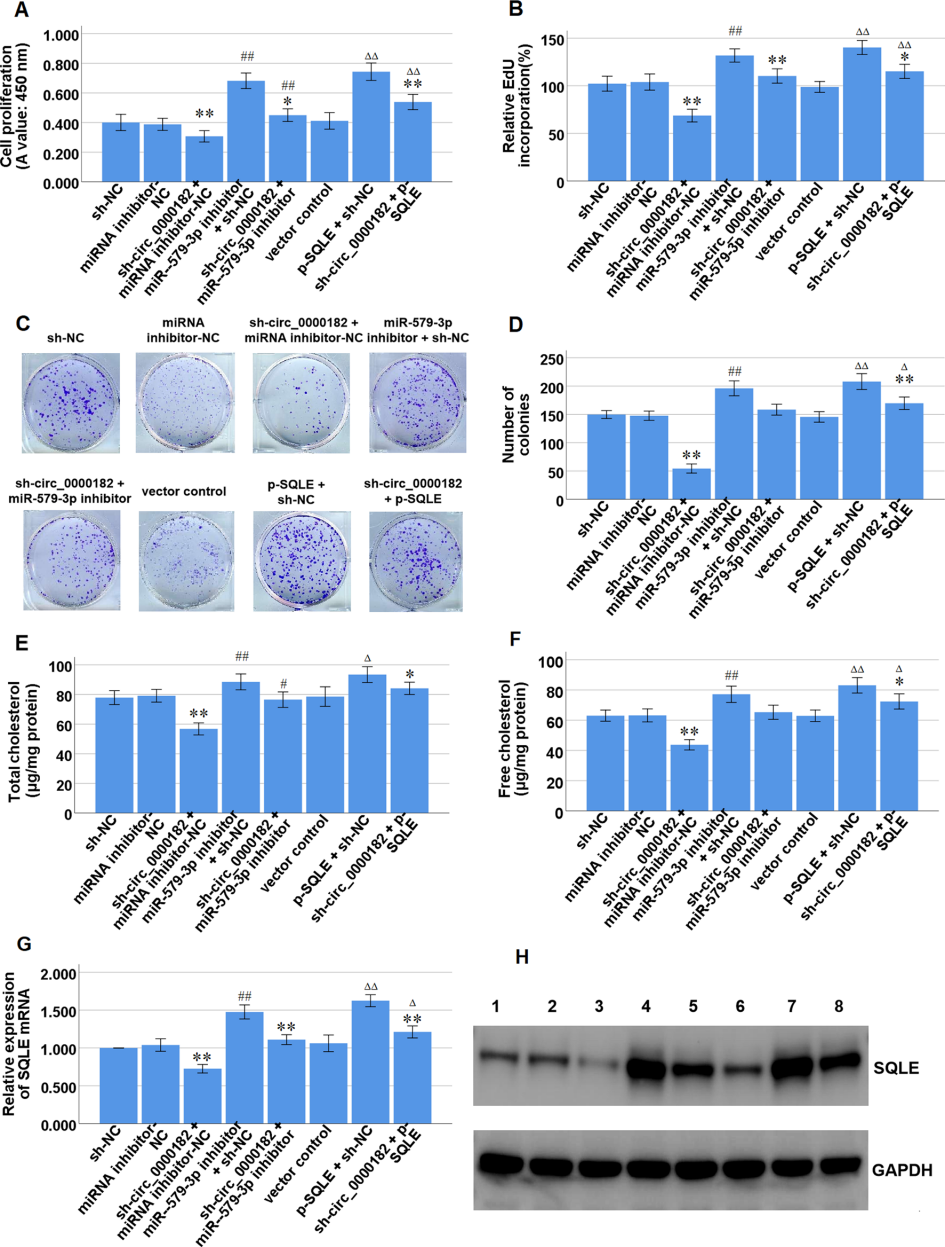
Effects of miR-579-3p inhibition or SQLE over-expression on cell proliferation and cholesterol synthesis inhibited by circ_0000182 knockdown in BGC-823 cells. **A** The CCK-8 assay was performed to detect the cell proliferation following the indicated transfections in BGC-823 cells.** B** The EdU incorporation assay was performed to detect the cell proliferation following the indicated transfections in BGC-823 cells. **C, D** The colony formation assay was performed to detect the cell proliferation following the indicated transfections in BGC-823 cells. **E, F** The commercial kits was used to detect TC and FC levels following the indicated transfections in BGC-823 cells. **G** qRT-PCR analysis of SQLE mRNA expression following the indicated transfections in BGC-823 cells. **H** Western blot analysis of SQLE protein expression following the indicated transfections in BGC-823 cells. Lane 1, sh-NC; Lane 2, miRNA inhibitor-NC; Lane 3, sh-circ_0000182 + miRNA inhibitor-NC; Lane 4, miR-579-3p inhibitor + sh-NC; Lane 5, sh-circ_0000182 + miR-579-3p inhibitor; Lane 6, vector control; Lane 7, p-SQLE + sh-NC; Lane 8, sh-circ_0000182 + p-SQLE. *P < 0.05, and **P < 0.01 *vs* sh-NC; ^#^*P* < 0.05, and ^##^*P* < 0.01 *vs* miRNA inhibitor-NC; ^Δ^*P* < 0.05, and ^ΔΔ^*P* < 0.01 *vs* vector control

Rescue assays were carried out by decreasing miR-579-3p expression via miR-579-3p inhibitor or increasing SQLE expression via p-SQLE in BGC-823 cells with circ_0000182 knockdown via sh-circ_0000182#1. The results of CCK-8, EdU incorporation and colony formation assays manifested that the cell proliferation of BGC-823 cells was significantly inhibited by circ_0000182 knockdown, and then it was partially reversed by miR-579-3p inhibition or SQLE over-expression (Fig. 6A–D). Meanwhile, the TC and FC levels in BGC-823 cells were significantly decreased by circ_0000182 knockdown, and then they were partially reversed by miR-579-3p inhibition or SQLE over-expression (Fig. 6E, F). The data indicated that miR-579-3p inhibition or SQLE over-expression in BGC-823 cells partially recovered cell proliferation and cholesterol synthesis, which was inhibited by circ_0000182 knockdown. Consistently, SQLE mRNA and protein expression levels were significantly decreased by circ_0000182 knockdown, and then they were partially reversed by miR-579-3p inhibition or SQLE over-expression (Fig. 6G, H). The data indicated that miR-579-3p inhibition or SQLE over-expression in BGC-823 cells partially recovered SQLE mRNA and protein expressions, which were inhibited by circ_0000182 knockdown. Taken together, these data suggest that circ_0000182 promotes cell proliferation and cholesterol synthesis of BGC-823 cells via miR-579-3p/SQLE axis.

## Discussion

Currently, although a number of circRNAs, functioning as oncogenes or tumor suppressors, have been reported to be involved in STAD progression [28–31], no studies have so far addressed circ_0000182’s role in STAD. Furthermore, there has been a few recent studies presenting that circRNAs have a strong ability to participate in regulating cholesterol metabolism in non-tumor cells, such as macrophages and aortic endothelial cells [20, 21]. However, there are few studies on circRNAs in cholesterol metabolism of tumors. In this study, we observed that circ_0000182 was increased in STAD. Furthermore, our present data validated the promotive role of circ_0000182 on cholesterol synthesis and proliferation in STAD cells. These findings were announced to be the first-hand document that the oncogenic factor of circ_0000182 in STAD provides a new perspective regarding tumor diagnosis and treatment.

Abnormal activation of cholesterol metabolism is now recognized as the prominent hallmark of human tumors, including STAD [9–12]. Interestingly, SQLE, a key rate-limiting enzyme in cholesterol synthesis, has long been considered a proto-oncogene because of its high copy number amplification and driving abnormal cholesterol metabolism in various type of tumors [32–35]. Recently, highly expressed SQLE has been shown to promote the cell proliferation of colorectal cancer (CRC) [35], prostate cancer (PCa) [36] and small cell lung cancer (SCLC) [37]. Similar to these former reports, our data displayed that SQLE expression was apparently elevated in STAD tissues, and over-expressed SQLE promoted cholesterol synthesis and proliferation in STAD cells, suggesting that SQLE functions as an oncogene in STAD, and SQLE-mediated cholesterol metabolism is involved in STAD progression. Furthermore, an animal study illustrated that SQLE inhibition with NB-598 in SCLC cells decreased cholesterol synthesis, and restrained the tumor growth in vivo [37]. Therefore, targeting SQLE-mediated cholesterol synthesis may be an effective strategy for controlling the occurrence and development of tumors. Here, we further found that circ_0000182 knockdown inhibited SQLE expression, cholesterol synthesis and proliferation, and meanwhile SQLE over-expression partly reversed the inhibitory effects of circ_0000182 knockdown. Therefore, these results suggest that circ_0000182 exerts an oncogene effect by up-regulating SQLE expression, and is a potential diagnostic marker and therapeutic target for STAD diagnosis and treatment.

It has been revealed that miRNAs are involved in the occurrence and development of STAD [38]. Interestingly, SQLE was found to be a target of miR-205 in PCa, and SQLE expression was negatively regulated by over-expressed miR-205 in PCa cells [36]. Furthermore, miRNAs has been identified to accomplish their biological functions by directly binding to the 3′-UTR of downstream target mRNAs [39]. Here, we found that miR-579-3p expression had a negative correlation with SQLE mRNA expression in STAD tissues, and over-expressed miR-579-3p inhibited SQLE mRNA expression in STAD cells. Furthermore, we confirmed that SQLE mRNA 3′-UTR contained a binding site for miR-579-3p in STAD cells. These results suggest that miR-579-3p down-regulates SQLE expression in STAD cells by directly targeting SQLE mRNA 3′-UTR. Subsequently, through bioinformatics analysis and experimental validation, we found that circ_0000182, a newly discovered circRNA, had a targeted binding site with miR-579-3p in STAD. Some circRNAs are abundant in STAD and have been proved to exert vital roles in STAD progression [17]. Furthermore, circRNAs were reported to participate in regulation of cholesterol metabolism by reducing cholesterol efflux from macrophages and promoting cholesterol synthesis in aortic endothelial cells [20, 21]. Here, we confirmed circular characteristics of circ_0000182 and elevated circ_0000182 expression in STAD. And we further found that over-expressed circ_0000182 promoted cholesterol synthesis and proliferation in STAD cells. Based on these evidences, we confirmed that circ_0000182 functioned as an oncogene by participating in regulation of cholesterol synthesis in STAD.

**Fig. 7:**
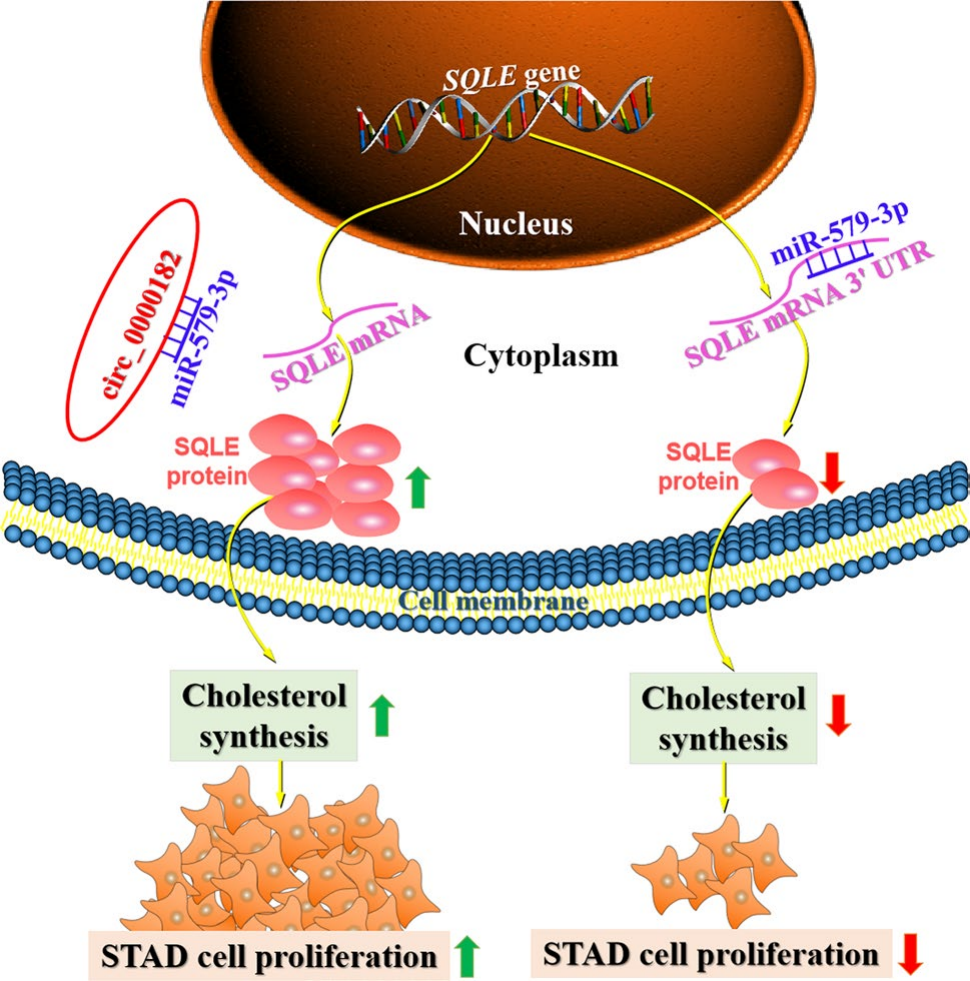
Schematic model demonstrates the results of the study. Circ_0000182 functions as a sponge of miR-579-3p by adsorbing miR-579-3p in the cytoplasm, and then miR-579-3p is prevented from binding to SQLE mRNA 3'-UTR, which leads to increased expression of SQLE. Due to the increased SQLE expression in STAD cells, cholesterol synthesis is increased, and then cell proliferation is promoted. However, when the specific adsorption of circ_0000182 is lacking, miR-579-3p binds to SQLE mRNA 3'-UTR, and the expression of SQLE is inhibited, thereby leading to a decrease in cholesterol synthesis and cell proliferation in STAD cells

CeRNA networks based on circRNAs have an impact on the occurrence and development of gastrointestinal cancers [40]. Furthermore, it has been found that circRNAs acted as ceRNAs for miRNAs to regulate proliferation of STAD cells [28, 31]. Interestingly, lncRNA‐TTN‐AS1 was found to act as a ceRNA for miR‐133b to regulate the expression of SQLE in pancreatic cancer cells [41]. Here, we demonstrated that over-expressed circ_0000182 down-regulated miR-579-3p expression and up-regulated SQLE expression in STAD, and confirmed that miR-579-3p directly interacted with circ_0000182 or SQLE mRNA 3'-UTR in STAD cells. Furthermore, we subsequently investigated the relationship between circ_0000182 and cholesterol metabolism, and found that circ_0000182 participated in promoting cholesterol synthesis and proliferation in STAD. Combined with the regulatory relationship between miR-579-3p and SQLE and the adsorption relationship between circ_0000182 and miR-579-3p, these results suggest that circ_0000182 acts as a ceRNA for miR‐579-3p to up-regulate SQLE expression, resulting in promoted cholesterol synthesis and proliferation in STAD cells (Fig. 7).

## Conclusion

In summary, we demonstrated that circ_0000182 acted as tumor promotion factor in STAD, and served as a ceRNA to counteract miR-579-3p-mediated SQLE suppression, thus promoting cholesterol synthesis and proliferation in STAD. Our present study not only identifies circ_0000182, a newly discovered circRNA, in STAD and its roles in STAD progression and cholesterol metabolism, but also provides a potential diagnostic marker and therapeutic target for STAD.

## Supplementary Information

**Additional file 1: Figure S1.** Original image of Western blotting (Fig.6H) showed the expression of SQLE.

**Additional file 2: Figure S2.** Original image of Western blotting (Fig.6H) showed the expression of GAPDH.

**Additional file 3: Table S1.** SQLE is predicted to be targeted by 34 miRNAs in miRDB.

**Additional file 4: Table S2.** The binding of miR-579-3p to SQLE mRNA 3' UTR predicted in TargetScanHuman database.

**Additional file 5: Table S3.** The binding of circ_0000182 to miR-579-3p predicted in Circular RNA Interactome.

## Data Availability

The datasets used and/or analyzed during the current study are available from the corresponding authors on reasonable request.

## Abbreviations

circRNA: Circular RNA
STAD: Stomach adenocarcinoma
SQLE: Squalene epoxidase
miR-579-3p: Has-miR-579-3p
circ_0000182: Hsa_circ_0000182
qRT-PCR: Quantitative real-time PCR
CCK-8: Cell counting kit-8
RIP: RNA immunoprecipitation
IHC: Immunohistochemistry
FLVCR1: Feline leukemia virus subgroup C cellular receptor 1
ceRNA: Competing endogenous RNA
IARC: International agency for research on cancer
WHO: World health organization
ESCC: Esophageal squamous cell carcinoma
CRC: Colorectal cancer
SCLC: Small cell lung cancer
GEPIA: Gene expression profiling interactive analysis
RISC: RNA induced silencing complex
TC: Total cholesterol
FC: Free cholesterol
miRNA: MicroRNA
non-coding RNA: NcRNAs
OSR: Overall survival rate
CVD: Cardiovascular disease
shRNA: Short hairpin RNA
UTR: Untranslated region
FBS: Fetal bovine serum
TNM: Tumor-node-metastasis
PCa: Prostate cancer
APOA1: Apolipoprotein A1
HDL-C: High-density lipoprotein cholesterol
