# Supplementary figures and images for: Circ_0000182 promotes cholesterol synthesis and proliferation of stomach adenocarcinoma cells by targeting miR-579-3p/SQLE axis
Source: Discov Oncol. 2023 Feb 20;14:22. doi: 10.1007/s12672-023-00630-5 (PMC9941389; doi:10.1007/s12672-023-00630-5)

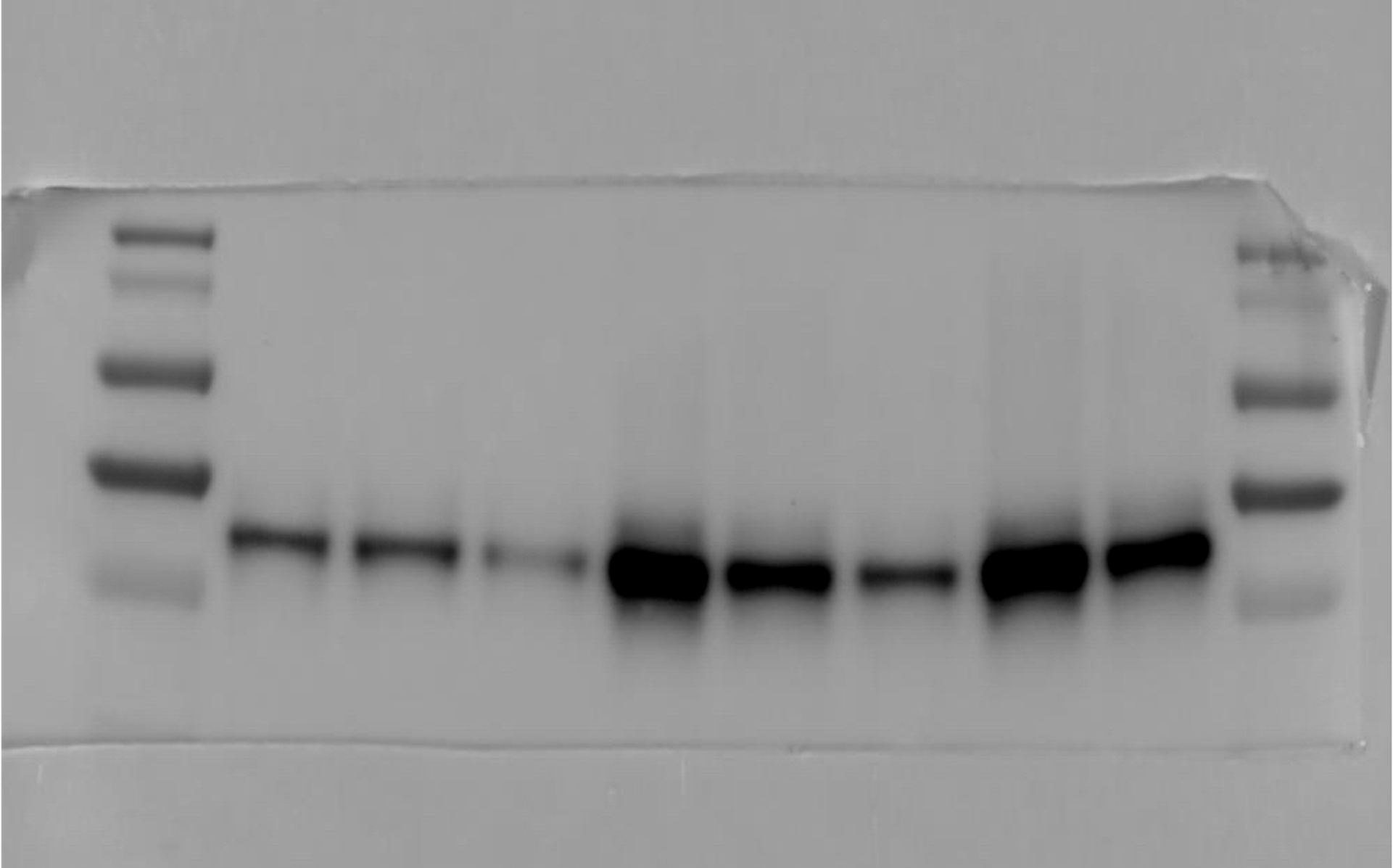

Supplement: Supplementary file 1 — Additional file 1: Figure S1. Original image of Western blotting (Fig.6H) showed the expression of SQLE. [file 12672_2023_630_MOESM1_ESM.tif]

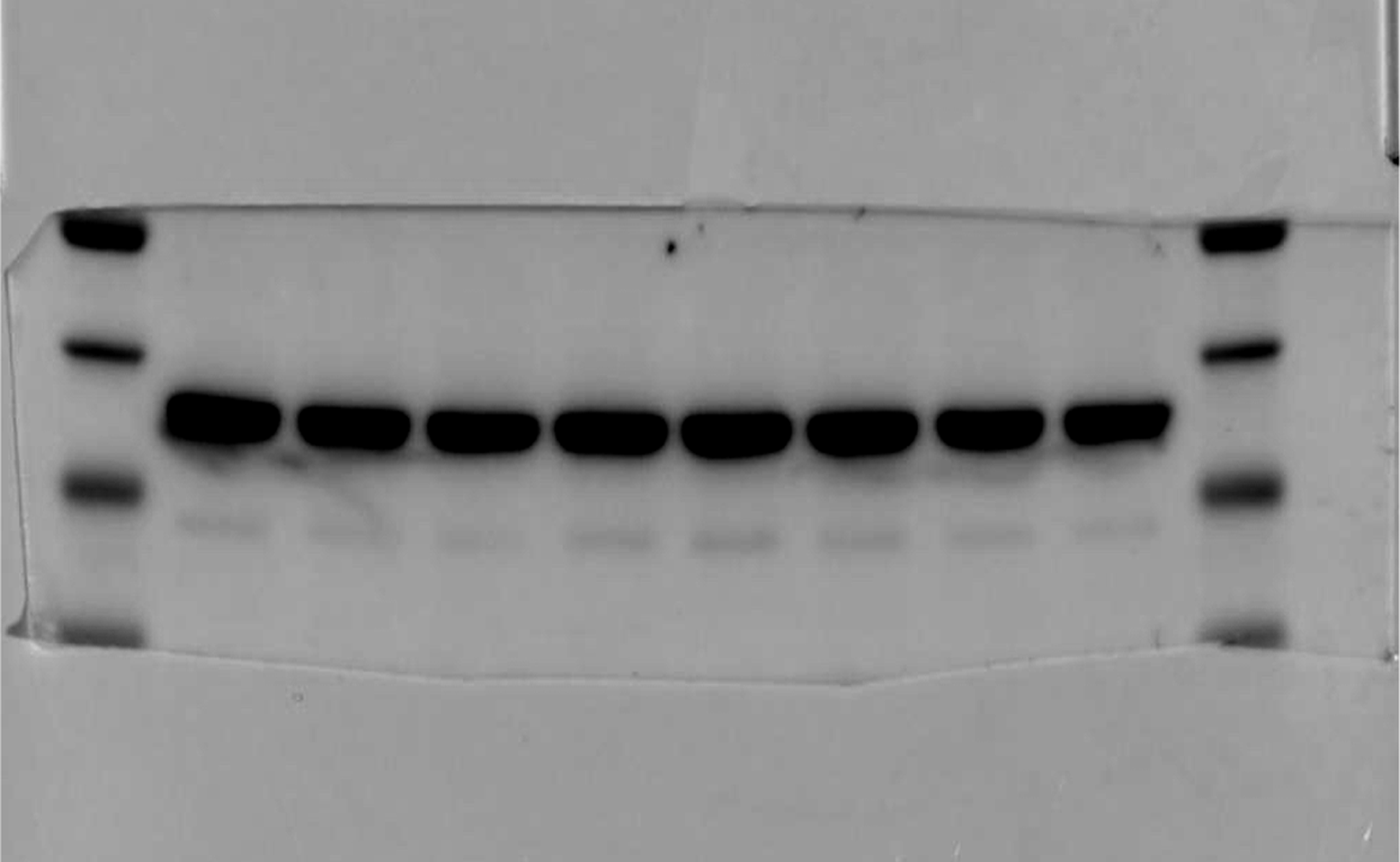

Supplement: Supplementary file 2 — Additional file 2: Figure S2. Original image of Western blotting (Fig.6H) showed the expression of GAPDH. [file 12672_2023_630_MOESM2_ESM.tif]
