# Supplementary material for: Circ_0000182 promotes cholesterol synthesis and proliferation of stomach adenocarcinoma cells by targeting miR-579-3p/SQLE axis
Source: Discov Oncol. 2023 Feb 20;14:22. doi: 10.1007/s12672-023-00630-5 (PMC9941389; doi:10.1007/s12672-023-00630-5)
Supplement: Supplementary file 3 — Additional file 3: Table S1. SQLE is predicted to be targeted by 34 miRNAs in miRDB. [file 12672_2023_630_MOESM3_ESM.docx]

**Table S1.** SQLE is predicted to be targeted by 34 miRNAs in miRDB

| **Target Rank** | **Target Score** | **miRNA Name** | **Gene Symbol** | **Gene Description** |
| --- | --- | --- | --- | --- |
| 1 | 96 | [hsa-miR-153-5p](https://mirdb.org/cgi-bin/mature_mir.cgi?name=hsa-miR-153-5p) | SQLE | squalene epoxidase |
| 2 | 96 | [hsa-miR-5696](https://mirdb.org/cgi-bin/mature_mir.cgi?name=hsa-miR-5696) | SQLE | squalene epoxidase |
| 3 | 96 | [hsa-miR-664b-3p](https://mirdb.org/cgi-bin/mature_mir.cgi?name=hsa-miR-664b-3p) | SQLE | squalene epoxidase |
| **4** | **96** | **[hsa-miR-579-3p](https://mirdb.org/cgi-bin/mature_mir.cgi?name=hsa-miR-579-3p)** | **SQLE** | **squalene epoxidase** |
| 5 | 94 | [hsa-miR-1250-3p](https://mirdb.org/cgi-bin/mature_mir.cgi?name=hsa-miR-1250-3p) | SQLE | squalene epoxidase |
| 6 | 92 | [hsa-miR-877-3p](https://mirdb.org/cgi-bin/mature_mir.cgi?name=hsa-miR-877-3p) | SQLE | squalene epoxidase |
| 7 | 89 | [hsa-miR-133a-3p](https://mirdb.org/cgi-bin/mature_mir.cgi?name=hsa-miR-133a-3p) | SQLE | squalene epoxidase |
| 8 | 89 | [hsa-miR-133b](https://mirdb.org/cgi-bin/mature_mir.cgi?name=hsa-miR-133b) | SQLE | squalene epoxidase |
| 9 | 85 | [hsa-miR-3127-3p](https://mirdb.org/cgi-bin/mature_mir.cgi?name=hsa-miR-3127-3p) | SQLE | squalene epoxidase |
| 10 | 85 | [hsa-miR-6756-3p](https://mirdb.org/cgi-bin/mature_mir.cgi?name=hsa-miR-6756-3p) | SQLE | squalene epoxidase |
| 11 | 83 | [hsa-miR-4436b-5p](https://mirdb.org/cgi-bin/mature_mir.cgi?name=hsa-miR-4436b-5p) | SQLE | squalene epoxidase |
| 12 | 79 | [hsa-miR-4699-3p](https://mirdb.org/cgi-bin/mature_mir.cgi?name=hsa-miR-4699-3p) | SQLE | squalene epoxidase |
| 13 | 75 | [hsa-miR-5579-5p](https://mirdb.org/cgi-bin/mature_mir.cgi?name=hsa-miR-5579-5p) | SQLE | squalene epoxidase |
| 14 | 74 | [hsa-miR-372-5p](https://mirdb.org/cgi-bin/mature_mir.cgi?name=hsa-miR-372-5p) | SQLE | squalene epoxidase |
| 15 | 71 | [hsa-miR-4735-5p](https://mirdb.org/cgi-bin/mature_mir.cgi?name=hsa-miR-4735-5p) | SQLE | squalene epoxidase |
| 16 | 70 | [hsa-miR-4422](https://mirdb.org/cgi-bin/mature_mir.cgi?name=hsa-miR-4422) | SQLE | squalene epoxidase |
| 17 | 67 | [hsa-miR-190a-3p](https://mirdb.org/cgi-bin/mature_mir.cgi?name=hsa-miR-190a-3p) | SQLE | squalene epoxidase |
| 18 | 66 | [hsa-miR-3613-3p](https://mirdb.org/cgi-bin/mature_mir.cgi?name=hsa-miR-3613-3p) | SQLE | squalene epoxidase |
| 19 | 65 | [hsa-miR-12123](https://mirdb.org/cgi-bin/mature_mir.cgi?name=hsa-miR-12123) | SQLE | squalene epoxidase |
| 20 | 63 | [hsa-miR-8084](https://mirdb.org/cgi-bin/mature_mir.cgi?name=hsa-miR-8084) | SQLE | squalene epoxidase |
| 21 | 62 | [hsa-miR-548am-3p](https://mirdb.org/cgi-bin/mature_mir.cgi?name=hsa-miR-548am-3p) | SQLE | squalene epoxidase |
| 22 | 62 | [hsa-miR-548j-3p](https://mirdb.org/cgi-bin/mature_mir.cgi?name=hsa-miR-548j-3p) | SQLE | squalene epoxidase |
| 23 | 62 | [hsa-miR-548ah-3p](https://mirdb.org/cgi-bin/mature_mir.cgi?name=hsa-miR-548ah-3p) | SQLE | squalene epoxidase |
| 24 | 62 | [hsa-miR-548x-3p](https://mirdb.org/cgi-bin/mature_mir.cgi?name=hsa-miR-548x-3p) | SQLE | squalene epoxidase |
| 25 | 62 | [hsa-miR-3184-3p](https://mirdb.org/cgi-bin/mature_mir.cgi?name=hsa-miR-3184-3p) | SQLE | squalene epoxidase |
| 26 | 62 | [hsa-miR-548aq-3p](https://mirdb.org/cgi-bin/mature_mir.cgi?name=hsa-miR-548aq-3p) | SQLE | squalene epoxidase |
| 27 | 62 | [hsa-miR-548aj-3p](https://mirdb.org/cgi-bin/mature_mir.cgi?name=hsa-miR-548aj-3p) | SQLE | squalene epoxidase |
| 28 | 62 | [hsa-miR-548ae-3p](https://mirdb.org/cgi-bin/mature_mir.cgi?name=hsa-miR-548ae-3p) | SQLE | squalene epoxidase |
| 29 | 61 | [hsa-miR-6881-3p](https://mirdb.org/cgi-bin/mature_mir.cgi?name=hsa-miR-6881-3p) | SQLE | squalene epoxidase |
| 30 | 60 | [hsa-miR-205-5p](https://mirdb.org/cgi-bin/mature_mir.cgi?name=hsa-miR-205-5p) | SQLE | squalene epoxidase |
| 31 | 55 | [hsa-miR-5011-5p](https://mirdb.org/cgi-bin/mature_mir.cgi?name=hsa-miR-5011-5p) | SQLE | squalene epoxidase |
| 32 | 52 | [hsa-miR-6083](https://mirdb.org/cgi-bin/mature_mir.cgi?name=hsa-miR-6083) | SQLE | squalene epoxidase |
| 33 | 50 | [hsa-miR-4279](https://mirdb.org/cgi-bin/mature_mir.cgi?name=hsa-miR-4279) | SQLE | squalene epoxidase |
| 34 | 50 | [hsa-miR-4433b-5p](https://mirdb.org/cgi-bin/mature_mir.cgi?name=hsa-miR-4433b-5p) | SQLE | squalene epoxidase |
