# Supplementary material for: Circ_0000182 promotes cholesterol synthesis and proliferation of stomach adenocarcinoma cells by targeting miR-579-3p/SQLE axis
Source: Discov Oncol. 2023 Feb 20;14:22. doi: 10.1007/s12672-023-00630-5 (PMC9941389; doi:10.1007/s12672-023-00630-5)
Supplement: Supplementary file 4 — Additional file 4: Table S2. The binding of miR-579-3p to SQLE mRNA 3' UTR predicted in TargetScanHuman database. [file 12672_2023_630_MOESM4_ESM.docx]

**Table S2.** The binding of miR-579-3p to SQLE mRNA 3' UTR predicted in TargetScanHuman database

|  | **Predicted consequential pairing of target region (top) and miRNA (bottom)** | **Site type** | **Context++ score** | **Context++ score percentile** | **Weighted context++ score** | **Conserved branch length** | **P_CT_** | **Predicted relative K_D_** |
| --- | --- | --- | --- | --- | --- | --- | --- | --- |
| Position 193-199 of SQLE 3' UTR  [hsa-miR-579-3p](http://www.mirbase.org/cgi-bin/mirna_entry.pl?acc=hsa-miR-579-3p) | 5'     ...UUAAUUUGCAAUUUA**AAAUGAA**G...                           \|\|\|\|\|\|\|  3'       UUAGCGCCAAAUAUGG**UUUACUU** | 7mer-A1 | -0.09 | 93 | -0.09 | 0.043 | N/A | -2.713 |
| Position 325-331 of SQLE 3' UTR  [hsa-miR-579-3p](http://www.mirbase.org/cgi-bin/mirna_entry.pl?acc=hsa-miR-579-3p) | 5'   ...UGGGACAUGCAAAUA**AAAUGAA**G...                         \|\|\|\|\|\|\|  3'    UUAGCGCCAAAUAUGG**UUUACUU** | 7mer-A1 | -0.10 | 93 | -0.09 | 0.031 | N/A | -2.912 |
