# Supplementary material for: Circ_0000182 promotes cholesterol synthesis and proliferation of stomach adenocarcinoma cells by targeting miR-579-3p/SQLE axis
Source: Discov Oncol. 2023 Feb 20;14:22. doi: 10.1007/s12672-023-00630-5 (PMC9941389; doi:10.1007/s12672-023-00630-5)
Supplement: Supplementary file 5 — Additional file 5: Table S3. The binding of circ_0000182 to miR-579-3p predicted in Circular RNA Interactome. [file 12672_2023_630_MOESM5_ESM.docx]

**Table S3.** The binding of circ_0000182 to miR-579-3p predicted in Circular RNA Interactome

| **CircRNA**  **Mirbase ID** | **CircRNA (Top) - miRNA (Bottom) pairing** | **Site Type** | **CircRNA Start** | **CircRNA End** | **3' pairing** | **local AU** | **position** | **TA** | **SPS** | **context+ score** | **context+ score percentile** |
| --- | --- | --- | --- | --- | --- | --- | --- | --- | --- | --- | --- |
| [circ_0000182](http://www.circbase.org/cgi-bin/singlerecord.cgi?id=hsa_circ_0000182) (5' ... 3')  [miR-579](http://www.mirbase.org/cgi-bin/mirna_entry.pl?acc=hsa-miR-579)-3p  (3' ... 5') | UCAACG**UU**AUUAAAU**CAAAUGA**U         \|\|\|     \|\|\|\|\|\|\|\|  UUAGCGC**CAA**AUAUG----**GUUUACU**U | 7mer-m8 | 331 | 337 | -0.016 | -0.114 | -0.045 | 0.041 | 0.068 | -0.186 | 99 |
